# Associations between glucocorticoid use and major adverse cardiovascular events in patients with prostate cancer receiving antiandrogen: a retrospective cohort study

**DOI:** 10.1038/s41391-024-00889-x

**Published:** 2024-09-10

**Authors:** Jeffrey Shi Kai Chan, Yan Hiu Athena Lee, Chi Ho Leung, David Ka Wai Leung, Edward Christopher Dee, Kenrick Ng, Gary Tse, Chi Fai Ng

**Affiliations:** 1 Cardio-Oncology Research Unit, Cardiovascular Analytics Group, PowerHealth Research Institute, Hong Kong, China; 2 SH Ho Urology Centre, Department of Surgery, Faculty of Medicine, The Chinese University of Hong Kong, Hong Kong, China; 3 Department of Radiation Oncology, Memorial Sloan Kettering Cancer Center, New York, NY USA; 4 Department of Medical Oncology, Barts Cancer Centre, London, UK; 5 Tianjin Key Laboratory of Ionic-Molecular Function of Cardiovascular Disease, Department of Cardiology, Tianjin Institute of Cardiology, Second Hospital of Tianjin Medical University, Tianjin, 300211 China; 6 Kent and Medway Medical School, Canterbury, Kent CT2 7NT UK; 7 School of Nursing and Health Sciences, Hong Kong Metropolitan University, Hong Kong, China

**Keywords:** Cancer therapy, Prostate cancer, Outcomes research

## Abstract

**Background:**

Prednisolone/prednisone coadministration with abiraterone may explain abiraterone-related increase in cardiovascular risk. We explored this postulation and glucocorticoid’s association with cardiovascular risk.

**Methods:**

Patients with prostate cancer on androgen deprivation therapy and enzalutamide, or abiraterone with 5 mg (ABI + P5) or 10 mg (ABI + P10) daily total prednisolone/prednisone were followed up for major adverse cardiovascular events (MACE).

**Results:**

We analyzed 933 patients. ABI + P10, but not enzalutamide, had higher risk of MACE than ABI + P5. Cumulative glucocorticoid dose before enzalutamide/abiraterone initiation was associated with MACE.

**Conclusions:**

Prednisolone/prednisone coadministration with abiraterone likely contributed to abiraterone-related increased cardiovascular risk. Prevalent cumulative glucocorticoid dose was associated with cardiovascular risk.

## Introduction

Androgen receptor signaling inhibitors (ARSIs) are recommended for the treatment of metastatic castration-resistant prostate cancer [1]. However, these agents are associated with increased cardiovascular risks [2]. We previously observed higher cardiovascular risk in abiraterone users than enzalutamide users [3], with abiraterone’s requirement for prednisolone/prednisone coadministration postulated as a cause. This study thus aimed to explore this postulation and the association between glucocorticoid use and cardiovascular risk in these patients.

## Methods

This retrospective cohort study was approved by the Joint Chinese University of Hong Kong—New Territories East Cluster Clinical Research Ethics Committee and followed the Declaration of Helsinki.

The data source was described previously [3]. Briefly, we used the Clinical Data Analysis and Reporting System (CDARS), a population-based health records database of all patients who attend public hospitals/clinics in Hong Kong. CDARS is linked to the governmental death registry. Both have been used extensively in research [4].

Patients aged ≥18 years old with prostate cancer who received enzalutamide or abiraterone atop androgen deprivation therapy (gonadotrophin-releasing hormone agonists and antagonists, and bilateral orchidectomy) in Hong Kong between 1/12/1999 and 31/3/2021 were included. The following patients were excluded: (a) received both drugs simultaneously/separately, (b) with abiraterone initiated without glucocorticoids, (c) with prior stroke, myocardial infarction (MI), or heart failure (HF), (d) with enzalutamide initiated with any glucocorticoid, and (e) with abiraterone initiated with any glucocorticoid regimen that is not prednisolone/prednisone 5 mg daily, 5 mg twice daily, nor 10 mg daily. Exclusion criteria (d) and (e) were added to the ones used in our prior study [3] as these prescriptions were not standard for enzalutamide/abiraterone regimens and were likely for other indications.

Patients were followed up from enzalutamide/abiraterone initiation (“index”) until 30/9/2021 or death, whichever earlier. Per our prior study [3], the primary outcome was MACE, a composite of all-cause mortality, MI, HF, and stroke. As some studies suggested efficacy differences between enzalutamide and abiraterone [5], we included an alternatively defined MACE (MACE_alternative_) as a secondary outcome, defined as a composite of non-PCa-related mortality, MI, HF, and stroke. Outcome and covariate ascertainment have been described in our prior study [3].

The exposure groups (regimen at the start of follow-up) were enzalutamide, abiraterone with 5 mg daily total of prednisolone/prednisone (ABI + P5), and abiraterone with 10 mg daily total of prednisolone/prednisone (ABI + P10).

The association between exposure and the risk of MACE was modeled using multivariable Cox regression, while that for the cumulative incidence of MACE_alternative_ was modelled using multivariable Fine-Gray competing risk regression with PCa-related mortality as the competing event. All regressions were adjusted for pre-specified covariates as listed in Supplementary Table 1. Cumulative glucocorticoid dose at index (CGD) was analyzed as log-transformed prednisolone-equivalent dose (as ln(1 + [cumulative glucocorticoid dose]) to allow transformation of zeros) [6].

In a post-hoc sensitivity analysis, only enzalutamide was compared against ABI + P10, as these groups were more similar in PCa treatment-related covariates (e.g. pre-index ADT duration).

Two-sided *p* < 0.05 were considered statistically significant. All analyses were performed using Stata v16.1 (StataCorp LLC, United States).

## Results

Altogether, 933 patients were analyzed (enzalutamide: 392; ABI + P5: 92; ABI + P10: 449; Supplementary Table 1 and Supplementary Fig. 1). Over a median follow-up of 24.2 (interquartile range: 13.6–41.9) months, MACE occurred in 535 patients (57.3%), whilst MACE_alternative_ occurred in 278 (29.8%).

**Fig. 1:**
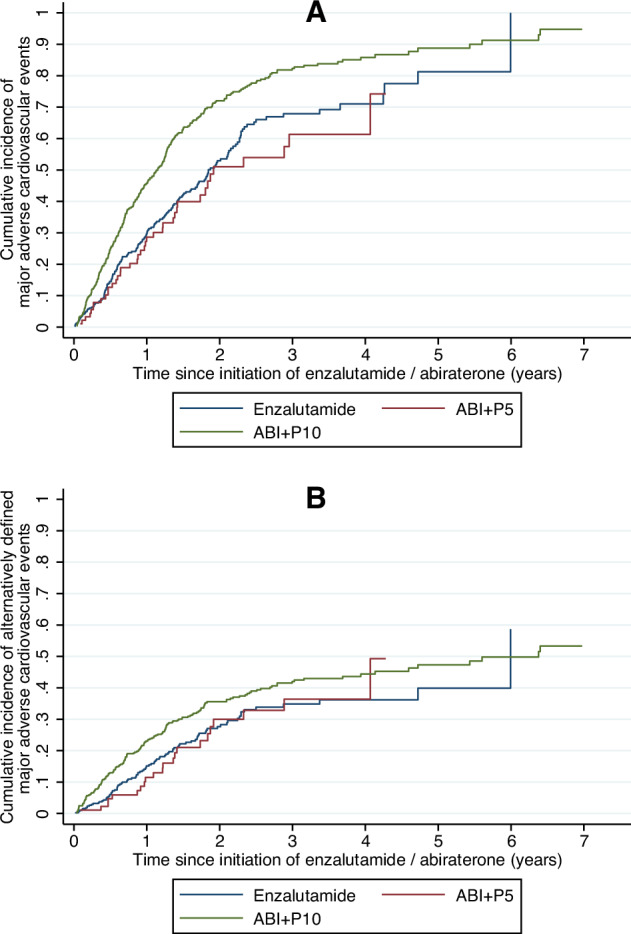
Cumulative incidence curves. Aalen-Johansen cumulative incidence curves of **A** major adverse cardiovascular events and **B** alternatively defined major adverse cardiovascular events, stratified by exposure groups (enzalutamide, abiraterone with 5 mg daily total of prednisolone/prednisone [ABI + P5], and abiraterone with 10 mg daily total of prednisolone/prednisone [ABI + P10]).

Compared to ABI + P5, ABI + P10 had higher risks of both MACE (adjusted hazard ratio [aHR] 1.90 [95% confidence interval: 1.34–2.68], *p* < 0.001; Fig. 1A) and MACE_alternative_ (adjusted subhazard ratio [aSHR] 1.53 [1.07–2.17], *p* = 0.019; Fig. 1B). Meanwhile, enzalutamide did not differ from ABI + P5 for both outcomes (MACE: aHR 1.31 [0.91–1.88], *p* = 0.144; MACE_alternative_: aSHR 1.06 [0.79–1.41], *p* = 0.700). Notably, a higher CGD was associated with higher risks of MACE (aHR 1.10 [1.05–1.16], *p* < 0.001) and MACE_alternative_ (aSHR 1.06 [1.03–1.10], *p* < 0.001).

In the sensitivity analysis (Supplementary Table 2), enzalutamide had significantly lower risks of MACE and MACE_alternative_. A higher CGD was associated with higher risks of both outcomes.

## Discussion

We observed that ABI + P10 was associated with higher cardiovascular risks than enzalutamide and ABI + P5 (the two of which did not differ significantly in risk) despite enzalutamide users having numerically more comorbidities, and that a higher CGD at index was associated with higher cardiovascular risks. Overall, these findings supported the postulation that prednisolone/prednisone coadministration with abiraterone contributed to abiraterone’s reported increase in cardiovascular risk compared to enzalutamide [3]. This was consistent with a previous study suggesting that high dose glucocorticoid (>10 mg prednisolone/prednisone) was associated with increased MI risk [7]. The lack of significant differences in cardiovascular risk between enzalutamide and ABI + P5 was also consistent with LATITUDE and ARCHES, which found no significant increase in cardiovascular events with abiraterone and enzalutamide, respectively, compared to placebo [8, 9]. It may be prudent for clinicians to carefully consider patients’ CGD and cardiovascular risk before considering ARSIs. Further research should be undertaken to explore glucocorticoid-free abiraterone regimens, with a phase 2 trial suggesting that such regimen may be feasible in selected patients [10].

This study has several limitations. First, there was no data on cancer staging or whether the patients had castration-resistant or hormone-sensitive disease, which may confound the findings. Secondly, this study’s observational nature predisposed to residual confounding and reverse causality. Thirdly, the impact of exposure duration on cardiovascular outcomes was not assessed, which is difficult in observational studies. Randomized controlled trials in this area may be warranted. Lastly, individual data adjudication was not possible; miscoding of events and covariates may, therefore, be possible.

## Conclusions

In patients with prostate cancer receiving androgen deprivation therapy with enzalutamide/abiraterone, prednisolone/prednisone coadministration with abiraterone likely contributed to abiraterone-related increase in cardiovascular risk. A higher prevalent CGD was strongly associated with higher cardiovascular risks.

## Supplementary information

Supplementary material

## Data Availability

All data underlying this study are available on reasonable request to the corresponding authors.
