# Supplementary material for: Associations between glucocorticoid use and major adverse cardiovascular events in patients with prostate cancer receiving antiandrogen: a retrospective cohort study
Source: Prostate Cancer Prostatic Dis. 2024 Sep 10;28(2):516–8. doi: 10.1038/s41391-024-00889-x (PMC12106070; doi:10.1038/s41391-024-00889-x)

SUPPLEMENTARY MATERIAL

Supplementary Table 1. Characteristics of included patients.

	Enzalutamide	Abiraterone + 5mg total daily prednisolone/prednisone	Abiraterone + 10mg total daily prednisolone/prednisone
Sample size	392	92	449
Age, years [IQR]	72 [66-78]	73 [65-78]	69 [64-76]
Type of ADT, N (%)			
Pharmacological only	235 (60.0)	78 (84.8)	256 (57.0)
Surgical only	87 (22.2)	7 (7.6)	105 (23.4)
Both pharmacological and surgical	70 (17.9)	7 (7.6)	88 (19.6)
Hypertension, N (%)	150 (38.3)	20 (21.7)	132 (29.4)
Ischaemic heart disease, N (%)	32 (8.2)	2 (2.2)	34 (7.6)
Chronic kidney disease, N (%)	8 (2.0)	0 (0)	10 (2.2)
Diabetes mellitus, N (%)	95 (24.2)	13 (14.1)	51 (11.4)
Dyslipidaemia, N (%)	92 (23.5)	9 (9.8)	56 (12.5)
Other malignancies, N (%)	20 (5.1)	6 (6.5)	24 (5.4)
Ever received radical prostatectomy, N (%)	99 (25.3)	22 (23.9)	97 (21.6)
Ever received radiotherapy, N (%)	67 (17.1)	15 (16.3)	111 (24.7)
Ever received chemotherapy, N (%)	168 (42.9)	28 (30.4)	237 (52.8)
Anticoagulant use, N (%)	154 (39.3)	32 (34.8)	181 (40.3)
Prostate-specific antigen, ng/mL [IQR]	59.4 [14.7-228.5]	34.4 [8.9-217.5]	107.2 [27.9-339.0]
ADT duration prior to the start of follow-up, months [IQR]	29.2 [17.0-52.7]	7.0 [1.4-24.2]	30.4 [16.3-52.2]
Cumulative prednisolone-equivalent glucocorticoid dose at the start of follow-up, mg [IQR]	0 [0-36.6]	6.1 [6.1-12.2]	36.6 [6.1-91.5]

ADT, androgen deprivation therapy. IQR, interquartile range.

Supplementary Table 2. Results of the sensitivity analysis where only patients who received enzalutamide were compared against patients who received abiraterone with 10mg total daily dose of prednisolone.

	MACE (aHR)	$MACE_{alternative}$ (aSHR)
Primary analysis	0.60 [0.51-0.70], p<0.001	0.60 [0.47-0.77], p<0.001
Secondary analysis (with adjustment for cumulative glucocorticoid dose at the start of follow-up instead of prior systemic glucocorticoid use)	0.69 [0.58-0.81], p<0.001 [1]	0.68 [0.53-0.86], p=0.002 [2]

aHR, adjusted hazard ratio. SHR, adjusted subhazard ratio. MACE, major adverse cardiovascular events. $MACE_{alternative}$, alternatively defined major adverse cardiovascular events.

[1] aHR for log-transformed cumulative glucocorticoid dose at the start of follow-up: 1.10 [1.04-1.16], p=0.001

[2] aSHR for log-transformed cumulative glucocorticoid dose at the start of follow-up: 1.06 [1.03-1.10], p<0.001

Supplementary Figure 1. Patient flow diagram. HF, heart failure. MI, myocardial infarction. PSA, prostate-specific antigen.

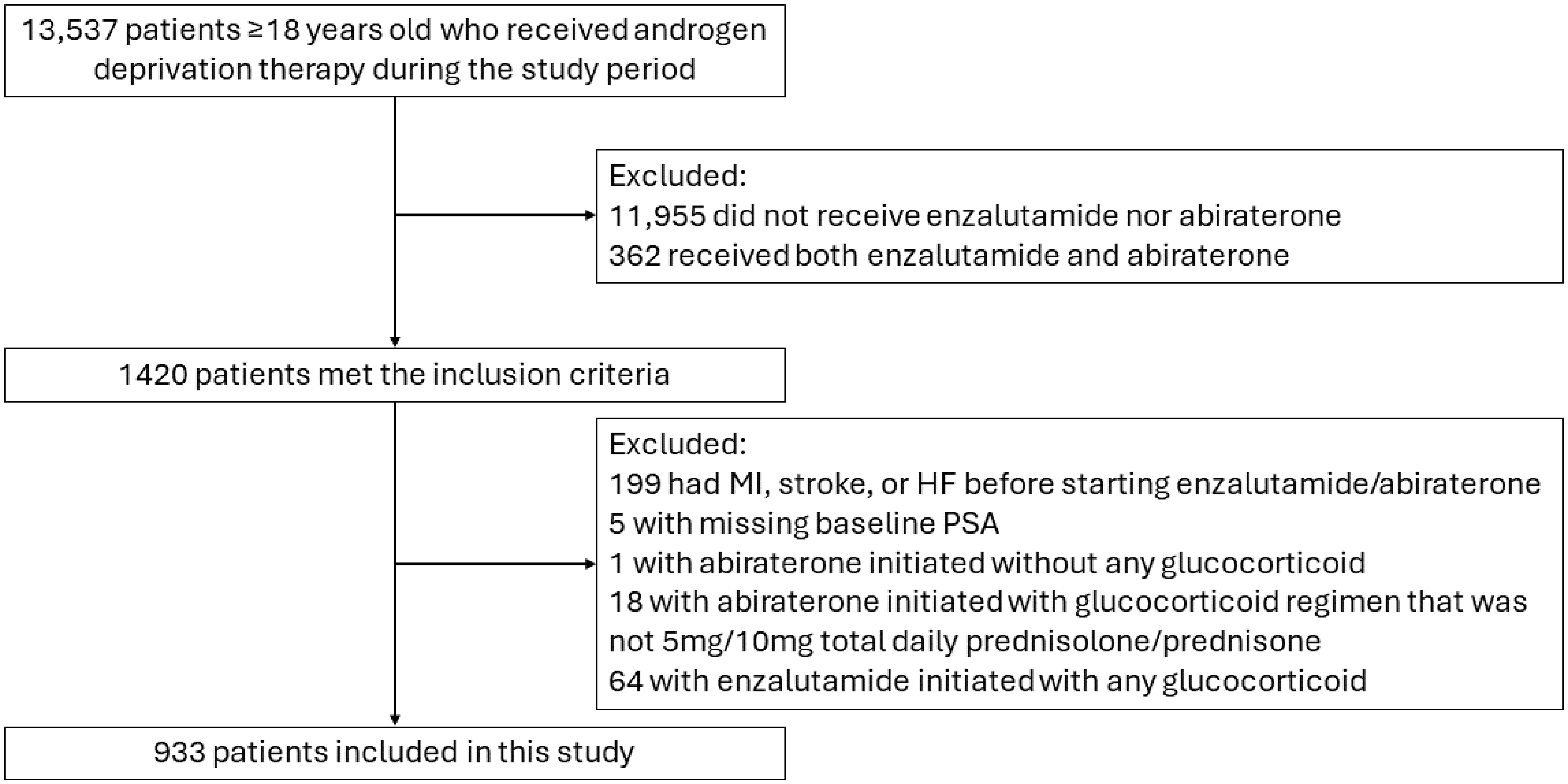

Supplement: Supplementary file 1 — Supplementary material [file 41391_2024_889_MOESM1_ESM.pdf]
